# Recent Advances in Surface Nanoengineering for Biofilm Prevention and Control. Part I: Molecular Basis of Biofilm Recalcitrance. Passive Anti-Biofouling Nanocoatings

**DOI:** 10.3390/nano10061230

**Published:** 2020-06-24

**Authors:** Paul Cătălin Balaure, Alexandru Mihai Grumezescu

**Affiliations:** 1 “Costin Nenitzescu” Department of Organic Chemistry, Faculty of Applied Chemistry and Materials Science, University Politehnica of Bucharest, G. Polizu Street 1-7, 011061 Bucharest, Romania; 2 Department of Science and Engineering of Oxide Materials and Nanomaterials, Faculty of Applied Chemistry and Materials Science, University Politehnica of Bucharest, G. Polizu Street 1-7, 011061 Bucharest, Romania

**Keywords:** nosocomial infections, antibiofilm coatings, molecular mechanisms of biofilm-associated antimicrobial resistance and tolerance, passive antiadhesive strategies, “fouling resistant”, “fouling release”

## Abstract

Medical device-associated infections are becoming a leading cause of morbidity and mortality worldwide, prompting researchers to find new, more effective ways to control the bacterial colonisation of surfaces and biofilm development. Bacteria in biofilms exhibit a set of “emergent properties”, meaning those properties that are not predictable from the study of free-living bacterial cells. The social coordinated behaviour in the biofilm lifestyle involves intricate signaling pathways and molecular mechanisms underlying the gain in resistance and tolerance (recalcitrance) towards antimicrobial agents as compared to free-floating bacteria. Nanotechnology provides powerful tools to disrupt the processes responsible for recalcitrance development in all stages of the biofilm life cycle. The present paper is a state-of-the-art review of the surface nanoengineering strategies currently used to design antibiofilm coatings. The review is structurally organised in two parts according to the targeted biofilm life cycle stages and molecular mechanisms intervening in recalcitrance development. Therefore, in the present first part, we begin with a presentation of the current knowledge of the molecular mechanisms responsible for increased recalcitrance that have to be disrupted. Further, we deal with passive surface nanoengineering strategies that aim to prevent bacterial cells from settling onto a biotic or abiotic surface. Both “fouling-resistant” and “fouling release” strategies are addressed as well as their synergic combination in a single unique nanoplatform.

## 1. Introduction. Pathogenic Biofilms: Characteristics and Developmental Stages

Healthcare-associated infections (HAIs) or nosocomial infections are acquired by 4.1 million patients in the European Union each year [1], the cost of treating these infections rising to about € 5.48 billion per year. The gravity of the situation is demonstrated by the number of deaths occurring as a direct consequence of HAIs, which is estimated to be at least 37,000 annually and by an additional number of 110,000 deaths due to other conditions that become complicated by HAIs [2]. According to estimates from the Centers for Disease Control and Prevention in the United States, biofilms are responsible for over 65% of hospital infections, a large proportion of these infections being linked to biofilm-invaded prostheses and indwelling medical devices [3]. Since modern medicine uses a plethora of implantable medical devices such as catheters, mechanical heart valves, cardiac pacemakers, coronary stents, cerebrospinal fluid shunts, joint prostheses, orthopedic fixation devices, biliary tract stents, breast implants, contact lens, dental prostheses, dental implants, and so on, colonisation of the surface of these devices by pathogenic biofilms creates a major public health problem.

The management of biofilm-related infections is a critical issue since the appropriate antimicrobial therapy is often ineffective due to the increased recalcitrance of biofilm-associated bacteria. The present review aims to highlight recent nanotechnology-based strategies for biofilm-infection control. We present nanostructured coatings that through appropriate manipulation of their surface physicochemical properties are able to repel bacteria, thereby impeding the attachment step and surface biofouling (passive strategies) or to kill bacteria on direct contact (active strategies), as well as nanocoatings whose bulk chemistry was modified so that they actively release antimicrobials or other compounds that are able to hamper biofilm formation or to increase susceptibility towards antimicrobials through various mechanisms such as degrading essential chemical constituents of the biofilm matrix or disrupting signaling pathways that are vital for biofilm formation, cooperative and collective behavior, and recalcitrance development. To this purpose, we structured our review into two parts. The first part includes a short presentation of biofilm developmental stages as well as a review of new insights into molecular mechanisms of biofilm recalcitrance towards currently used antimicrobials and ends with a discussion of the bio-inspired and biomimetic passive anti-biofilm nanocoatings. The second part deals with contact-killing and active releasing nanocoatings, combined switchable active-passive strategies, as well as smart nanocoatings capable of the on-demand release of their therapeutic payload in response to triggering environmental and physiological signals.

Infectious biofilms are sessile microbial aggregates exhibiting striking features, including here the expression of virulence factors that distinguish them from the same bacteria in isolation. In biofilms, bacterial cells are embedded in a sheltering extracellular polymeric matrix consisting of self-produced biomacromolecules, i.e., polysaccharides, proteins, and extracellular DNA (e-DNA) [4,5,6,7]. Some extracellular polymeric substances (EPS) such as e-DNA may also be derived from the lysis of the host immune cells (polymorphonuclear leukocytes). There are two types of bacterial biofilms: ones in which the multicellular aggregates attach to an abiotic or biotic surface and others in which the multicellular aggregates are not surface-attached but rather embedded in some host-derived materials such as mucus. The switch from the free-floating motile state to the sessile biofilm mode of growth is controlled by the intracellular second messenger cyclic-diguanosine monophosphate (c-di-GMP). In the transition from the planktonic to biofilm growth, c-di-GMP downregulates bacterial motility, switching it from flagellar movement to type IV pili twitching motility [8]. At the same time, c-di-GMP promotes surface attachment and biofilm development by the upregulation of encoding genes responsible for the production of adhesins and the EPS matrix [9].

One can distinguish three stages in the process of surface-associated biofilms formation: attachment, maturation, and dispersion [10]. The attachment step is governed by many forces, some of them attractive and others repulsive, which develop when bacterial cells approach a surface. The attractive forces receive support for overcoming the short distance (10–20 nm) repulsive forces from bacterial flagella, which eventually accomplish mechanical surface attachment. One can further describe the attachment step as a two-stage process: (*i*)—non-specific primary adhesion, which is a reversible process mediated by weak non-covalent intermolecular forces such as hydrophobic and van der Waal’s interactions and (*ii*)—specific, irreversible adhesion characterised by the production of specific proteins having the ability of covalent binding to the surface. The surface of an implanted medical device is chemically modified due to a biofouling process that starts soon after implantation resulting in surface deposition of a host-derived conditioning film. The conditioning film consists of small organic compounds and macromolecules, including proteins and polysaccharides. This is very important since the chemical nature of the surface of the conditioning film strongly influences subsequent bacterial cell adhesion [11]. Specific proteins produced in stage (*ii*) such as adhesins, autolysin/adhesins AtlE and Aae, the fibrinogen-binding protein Fbe/Sdrg, the fibronectin-binding protein Embp, and the lipase GehD attach to the human host proteins coating the implanted prosthesis or device. Once cells became irreversibly attached to the surface, biofilm maturation begins. The initial thin layer of cells grows thicker and thicker, developing into a mushroom or tower-shaped structure in the mature biofilm. In this developmental stage, complex biomolecular processes resulting in the upregulation of virulence factors and extracellular matrix constituents secretion and assembly occur. The final dispersion step is also critical for the biofilm life cycle, since it allows for the recolonisation of other available host sites. Several factors such as a lack of nutrients and intense competition might cause cells detachment, resulting in total or partial biofilm dispersal.

## 2. Molecular Mechanisms of Antimicrobial Agents Recalcitrance in Clinically Important Biofilms

Planktonic bacteria are more sensitive to antimicrobials than biofilm bacteria are. Killing biofilm-associated pathogens may require up to a 1000-fold increase of the antibiotic dose that usually kills free-floating bacteria [12,13], and most antibiotic-resistant infections are provoked by pathogenic bacteria in the biofilm mode of growth.

Biofilm resistance to antimicrobial agents may be defined as the capacity of microorganisms to continue to grow in the presence of bactericidal and bacteriostatic agents in a concentration higher than the minimum inhibitory concentration (MIC). On the other hand, tolerance to antimicrobials means that the microorganisms neither die nor grow but are able to survive in the presence of a bactericidal antimicrobial agent. In the biofilm mode of life, microorganisms develop both resistance and tolerance to antimicrobials. Therefore, a new term named recalcitrance was introduced to describe the decreased sensitivity of biofilms to antibiotics as a consequence of a complex mixture of resistance and tolerance mechanisms [7,14]. It is this acting in concert and the interplay of many self-defense mechanisms, some of them not fully understand nor investigated, which is still the subject of intense scientific debates, that makes the eradication of infectious biofilms such a difficult task to achieve. Consequently, a better understanding of various mechanisms contributing to biofilms antibiotics recalcitrance is crucial to identify and validate new drug targets and develop new significantly improved treatments that are able to overcome and/or bypass these mechanisms. The spectrum of molecular resistance and tolerance mechanisms elicited by biofilm bacteria is quite wide depending mainly on the particular bacterial species and strain, on the type of the antimicrobial agent, on the stage of biofilm maturation, and the particular growth conditions. However, five main types of determinants have been identified by researchers so far:

*(i)*  EPS matrix—especially the interaction of antimicrobials with its components;
*(ii)*  nutritional limitations and stress responses;
*(ii)*  persister cells;
*(iv)*  cell-to-cell communication in biofilms resulting in the enhanced expression of various specific genetic determinants of antibiotic resistance and tolerance. Transcriptomic and proteomic studies revealed sharp differences in the gene expression of genetically identical bacteria when they grow as aggregates in the biofilm mode of life and when they are isolated in the planktonic free-floating form. Some estimates indicate that as much as 40% of the genes of a bacterium may undergo up or downregulation and no less than half of the proteome is being differentially expressed in the transition from the planktonic to the biofilm state;
*(v)*  mutational (conventional) antibiotic resistance.

In the next paragraphs, some major insights into the molecular mechanisms of biofilms recalcitrance to antibiotics will be outlined.

### 2.1. Biofilm Matrix

Many authors presumed that the EPS matrix would hamper the penetration of antimicrobials within the biofilm depth. However, restricted antimicrobial diffusion seems to be of minor importance in determining recalcitrance, since even those antibiotics exhibiting easy penetrability into biofilms are still ineffective in killing biofilm-associated bacterial cells [15]. The EPS matrix is not just a simple physical barrier against penetration, but rather its chemical constituents play much more complex roles in recalcitrance development.

#### 2.1.1. Exopolysaccharides

The Main Polysaccharides Produced by Some Strains of *Pseudomonas* *aeruginosa* Biofilms are Psl and Pel.

Three pentasaccharides, namely D-glucose, D-mannose, and L-rhamnose are the structural repeating units of the Psl exopolysaccharide [13,16]. Controversial data regarding the role played by Psl in resistance to tobramycin have been reported [15,17,18]. It was suggested that Psl could physically confine antibiotics. The precise mechanism is still unclear, a charge-based entrapment of cationic antibiotics such as colistin and tobramycin being ruled out since Psl is uncharged.

Pel exopolysaccharide is another important structural component of the biofilm matrix of *P*. *aeruginosa*. Identical to Psl, it seems to provide protection against aminoglycoside antibiotics such as tobramycin and gentamicin, since biofilms lacking Pel showed increased susceptibility to these antibiotics [19]. Again, the recent finding that Pel is positively charged being composed of partially acetylated 1→4 glycosidic linkages of N-acetylgalactosamine and N-acetylglucosamine [20] ruled out the possibility of electrostatic sequestration of cationic antibiotics. It rather appears that instead of complexing the antibiotics, Pel is involved in the cross-linking of e-DNA in the biofilm stalk via ionic attractions, thereby reinforcing the structural integrity of the biofilm matrix. Since e-DNA itself plays a major role in resistance to antibiotics (as discussed below in Section 2.1.2), the mechanisms of acquired resistance mediated by e-DNA and Pel might be interconnected.

#### 2.1.2. e-DNA

The extracellular DNA found in the EPS matrix of most bacterial biofilms may be of either endogenous or exogenous origin. Endogenous e-DNA may be produced in a quorum sensing-mediated process (see Section 2.4), or it may derive from cell lysis within some biofilm subpopulations [21]. On the other hand, exogenous e-DNA is host-derived originating from the lysis of polymorphonuclear leukocytes at infection sites [22].

Since the sugar–phosphate backbone of DNA is deprotonated at physiological pH, the negative charge of the resulted polyanion is balanced by the binding of positively charged Mg^2+^ counterions [23]. This causes a local drop in Mg^2+^ concentration being the environmental signal that triggers activation of the PhoPQ and PmrAB two-component systems (see Section 2.4) in *P*. *aeruginosa* and *S. enterica* serovar Typhimurium biofilms [24,25,26]. At the same time, the slightly acidic microenvironment created by e-DNA accumulation is another signal synergistically contributing towards activation of the same PhoPQ and PmrAB two-component signal transduction pathways. These two-components systems upregulate the PA3552–3559 operon, which is responsible for the expression of genes-encoding enzymes able to modify the lipopolysaccharide (LPS) in the outer membrane of Gram-negative bacteria by adding amino arabinose to lipid A moiety [27]. This positively charged amino sugar could reduce the affinity of cationic antimicrobial peptides (AMP) to the bacteria resulting in increased bacterial resistance towards AMPs such as polymyxin B and colistin. According to Mulcahy, Charron-Mazenod, and Lewenza, the resistance of wild-type *P*. *aeruginosa* biofilms towards polymyxin B and colistin was increased by 4-fold and 8-fold respectively by adding e-DNA [27,28]. On the other hand, e-DNA addition has no effect concerning resistance increase for either antibiotic in a ΔPA3553 deletion mutant. Controversial results were obtained by different research groups investigating the role of the PA3552–3559 operon in promoting biofilm resistance to AMPs and aminoglycoside antibiotics [29], suggesting that other mechanisms besides protection of the outer bacterial membrane from the detrimental effect of AMPs might be involved.

Another potential mechanism underlying the role of e-DNA in the development of antibiotics resistance in biofilms is the horizontal transfer of antibiotic resistance genes (HGT) between bacterial cells (see also Section 2.5). Kouzel, Oldewurtel, and Maier provided convincing evidence for an efficient HGT between single-resistant bacteria within early *N. gonorrhoeae* biofilms [30].

#### 2.1.3. Extracellular Proteins

The biofilm matrices of various pathogenic bacteria such as *K.* *pneumoniae* and *P. aeruginosa* contain secreted antibiotic-modifying enzymes, namely β-lactamases, which can effectively inactivate β-lactam antibiotics. Bagge et al. [31] showed that the expression of ampC β-lactamase occurs in *P. aeruginosa* biofilms in response to exposure to β-lactam antibiotics such as imipenem and ceftazidime.

#### 2.1.4. The Viscous Environment of the EPS Matrix

Cairns et al. revealed an interesting mechanosensing mechanism that uses a positive feedback control loop coming into play during biofilm development in *Bacillus* *subtilis* chosen as the model Gram-positive bacterium [32,33]. These authors showed that the formation of the *B. subtilis* biofilm matrix is regulated by the bacterial flagellum acting as a mechanosensor that activates the DegS–DegU two-component signaling pathway. The secretion of exopolysaccharides during biofilm development renders the biofilm matrix more viscous. Increased viscosity restricts the rotation of the flagellum generating, in turn, an increased torque force on the flagellar motor. This mechanosensing mechanism is presumed to alter gene expression during the initial attachment step in biofilm development. In *B. subtilis* hampered flagellum, rotation triggers the biosynthesis of γ-poly glutamic acid (γ-PGA) in a DegS–DegU-mediated signal transduction process. Phosphorylated DegU activates the pgs operon, which is responsible for γ-PGA production, the main component of the EPS matrix of *B. subtilis*. So, increased biofilm matrix viscosity stops flagellar rotation, which in turn reinforces EPS matrix production and further raises viscosity. Thereby, sensing the EPS matrix viscosity by bacterial flagellum allows for a positive feedback loop regulation of EPS matrix production [33].

Last but not least, restricted bacterial motion due to biofilm matrix viscosity holds bacteria together and creates an optimal environment for horizontal gene transfer, including those DNA segments that encode virulence factors and antibiotic resistance genes [10].

### 2.2. Nutritional Limitation and Stress Responses

We saw that because of different gene expression, bacteria growing in biofilms are physiologically distinct from their free-floating counterparts. However, also bacteria living in different locations within the biofilm matrix appear as physiologically heterogeneous. That means that depending on their position within the biofilm, bacteria exhibit different phenotypes, metabolic activities, and antimicrobial tolerances. Heterogeneity arises as the biofilm grows. Great differences in the growth rate were observed between the outer fast-growing cells and cells located deeper within the biofilm, which exhibit nearly no metabolic activity [34]. The latter bacterial cells experience nutrient and oxygen shortages due to the local exhaustion of available nutritional resources, which prior to reaching deeper colonies are consumed by cells located next to the outer surface of the biofilm.

#### 2.2.1. Hypoxia and Reduced Growth Rate

Studies [35,36,37] showed that antibiotics targeting metabolic activities were ineffective in hypoxic biofilm areas, although they could effectively penetrate those zones, while steel kept their antimicrobial activity in other biofilm regions normally supplied with oxygen. The reverse effect was reported in *P. aeruginosa* biofilms exposed to colistin and aminoglycoside antibiotics [38]. It was suggested that the tolerance of *P. aeruginosa* biofilms to tobramycin is due to the reduction of the cell membrane potential in hypoxic zones. The intracellular uptake of aminoglycoside antibiotics requires the generation of an electrochemical gradient across the bacterial cell membrane, the so-named proton motive force (PMF), which is composed of an electrical potential gradient (ΔΨ) and a chemical gradient in proton concentration (ΔpH), which is acidic outside the plasma membrane and interior alkaline. Since at physiological pH aminoglycosides are polycationic molecules, their internalisation is driven by ΔΨ, which is interior negative. However, a critical transmembrane electrical potential threshold level of at least −155 mV [39,40,41,42] is needed for the intracellular uptake of aminoglycosides and further rRNA binding and protein synthesis inhibition. According to the chemiosmotic theory, the generation of ΔΨ is dependent on some key respiratory complexes of the electron transfer chain (ECT). Under hypoxic conditions, expression of the latter is downregulated [43], resulting in a reduction in ΔΨ and a consequently decreased intracellular uptake of aminoglycoside antibiotics.

A recent study [44] conducted by Tata et al. revealed another mechanism responsible for antibiotic resistance under hypoxic conditions. The genes encoding the MexCD-OprJ efflux pump were shown to be transcriptionally upregulated in *P*. *aeruginosa* biofilms grown under low-oxygen partial pressure. Overexpression of this pump renders the biofilm resistant to several classes of antibiotics, including fluoroquinolones, ß-lactams, tetracycline, chloramphenicol, macrolides, trimethoprim, and novobiocin.

#### 2.2.2. Oxidative Stress Responses

There are several pieces of evidence showing that a mechanism contributing to the bactericidal effect of antibiotics relies on an antibiotic-induced rise in reactive oxygen species (ROS), which might reach the lethal threshold [45,46,47,48,49,50]. Briefly, bactericidal antibiotics accelerate cellular respiration [45,51], leading to the overproduction of deleterious oxygen species (ROS) such as superoxide radical anion and hydrogen peroxide, which further disrupts iron homeostasis. Iron from the iron–sulphur clusters in redox enzymes of the respiratory chain is oxidised from Fe^2+^ to Fe^3+^ by hydrogen peroxide in a Fenton reaction, which generates increased amounts of hydroxyl radicals [52,53]. The latter contribute to cell death by producing oxidative damages to critical biomacromolecules such as DNA [51,52,54,55]. Bacteria counteract to reduce such damages using detoxifying enzymes, namely catalases, which decompose hydrogen peroxide. For instance, ciprofloxacin-induced overproduction of ROS in *P. aeruginosa* biofilms is diminished by KatA catalase, since a catalase-deficient ΔkatA mutant biofilm treated with ciprofloxacin produced higher amounts of hydroxyl radicals and ROS than the wild-type biofilm [47]. In addition, the viability of the mutant biofilm was decreased. As a concluding remark, the upregulation of catalase would contribute to the increased tolerance of *P. aeruginosa* biofilms towards ciprofloxacin. The stringent response triggered by nutrient starvation upregulates catalase activity [56,57] and decreases the production of pro-oxidant molecules such as 4-hydroxy-2-alkylquinolines (HAQs) [56,58,59], as discussed in the next section.

#### 2.2.3. The Stringent Response

The stringent response is an adaptive response allowing bacteria to survive under nutrient-deprived conditions. It was first observed under amino acid starvation conditions. Briefly, in response to amino acid starvation, bacteria adjust metabolic activities by rapidly initiating a metabolic switch from protein synthesis to amino acid synthesis. Ribosomal protein genes are downregulated, while amino acid biosynthetic genes are upregulated. The signaling pathway is initiated and controlled by the second messenger (p)ppGpp pair of guanosine penta and tetra phosphate, pppGpp and ppGpp, respectively. The intracellular concentration of the (p)ppGpp alarmone is regulated by RelA/SpoT Homologue (RSH) enzymes, which synthesise (p)ppGpp from ATP (Adenosine triphosphate) and GTP (Guanosine-5′-triphosphate)/GDP (Guanosine diphosphate) and hydrolyze (p)ppGpp to GTP/GDP and pyrophosphate, respectively. Unlike RelA, which has only synthetic activity stimulated by amino acid deprivation, SpoT is a bifunctional enzyme with both (p)ppGpp synthetic and hydrolytic activities acting as a signaling hub protein that senses and modulates responses to multiple nutritional stresses other than amino acid starvation such as fatty acid, carbon, iron, and phosphate-depleted environments [59,60,61]. Amino acid starvation leads to increased levels of deacylated tRNAs in the cytosol. The binding of these uncharged tRNAs to the ribosomal A-site is the signal that recruits RelA synthetase, which upon binding to the 50S ribosomal subunit is being activated and proceeds to (p)ppGpp alarmone synthesis. Further, the alarmone binds to RNA polymerase and modulates gene transcription [59,62], directing the cellular metabolic resources to amino acid synthesis and restoring the normal levels of aminoacylated tRNAs.

Since biofilm grown under nutrient starvation conditions is much less susceptible to a variety of differently acting antibiotics, the logical hypothesis that (p)ppGpp alarmone disrupts a killing mechanism common to diverse agents such as oxidative damage was launched and tested by Nguyen et al. [56]. In support of the above hypothesis and molecular mechanism underlying the stringent response [59] are the findings that (p)ppGpp alarmone-deficient biofilms formed by a *P*. *aeruginosa* ΔRelA ΔSpoT double-knockout strain were more susceptible than wild-type biofilms towards antibiotics from several different classes including ofloxacin, meropenem, colistin, and gentamicin [56].

The second messenger (p)ppGpp is not only a transcriptional regulator but is also involved in the emergence of bacterial persisters [63], as discussed below.

### 2.3. Persister Cells

Persisters are an antibiotic-tolerant differentiated phenotype of the wild type within a bacterial population [64]. Persister cells emerge stochastically, and their drug tolerance is non-inheritable. Therefore, persisters were presumed to be nongrowing, nondividing, metabolically inactive cells that arise in response to environmentally stressful conditions such as hypoxia, acidic pH, nutritional starvation, or antibiotic exposure being able to survive in this temporary dormant state until the stress is removed. After stress relieving, the persisters switch back to the actively growing state, thereby providing greater survival potential to the bacterial population. Whereas in a wild planktonic type *E. coli* population, the frequency of persister formation was found to be only 1 in a million, in stationary cultures and biofilms, an about 10^4^ fold increase of the persister subpopulation up to 1 in 100 was observed [65]. The molecular mechanism underlying the formation of persisters is largely unknown, and some authors even claimed that nongrowing cells in a bacterial population might not necessarily belong to the persister phenotype, while persisters might be metabolically active cells [66].

Nevertheless, some important insights into the mechanism of persister formation were gained through experimental studies ingeniously designed to overcome the inherent difficulties arising from the very low frequency of the persister phenotype. The main model underlying the genetic basis of persister formation involves the expression of toxin–antitoxin (TA) modules. The main role of TA systems is to regulate growth arrest and transition to the persistent state [60,62,63,64,65,67]. TA systems consist of a pair of genes within an operon. One gene encodes a protein toxin, while the second gene encodes the cognate antitoxin, which might be either a protein or a non-coding RNA molecule [65]. The toxin component interferes with a vital cellular process determining growth arrest. However, under normal growth conditions, the toxin is inactivated through binding to its complementary antitoxin. However, under stress conditions, the antitoxin is degraded, thereby leaving the toxin to achieve its full toxic effects freely. There are several types of TA systems (I–VI), the best studied being the type II TA system in which both toxin and antitoxin are proteins [65].

Moyed and Bertrand [68] were the first to demonstrate that bacterial persistence has a genetic basis through isolation of *E*. *coli* K12 mutants with vastly increased levels of persisters, i.e., high persistence or hip mutants. Further research showed that the hip operon consists of two genes, hipA and hipB. The wild-type hipA gene encodes a 440-residue protein toxin, whereas the upstream hipB gene encodes a smaller 88-residue DNA-binding protein [66]. Since hip B and hip A are on the same hipBA operon, they share the same promoter and are transcribed together. The hipB protein is a transcriptional repressor of the hipBA operon and downregulates its own promoter by cooperatively binding to four operators upstream of hipBA [69,70]. Hence, the expression of the hipBA operon is autoregulated. However, at the same time, the hipB protein acts as the antitoxin counterpart of the hipA protein toxin. Under normal growing conditions, both genes are expressed and the hipB antitoxin binds to the hipA toxin, forming a tight dimer complex and thereby neutralising the deleterious effects, i.e., growth arrest, of the latter. However, during cellular stress periods, the stress-induced Lon protease degrades the less stable hipB antitoxin, thereby freeing the toxin from its antitoxin counterpart [59,63,71,72,73,74]. In its unbound state, the hipA protein toxin acts as a kinase that phosphorylates and inhibits glutamyl–tRNA synthetase, resulting in an increased concentration of uncharged glutamyl tRNAs (tRNA^Glu^) [59,74,75]. In turn, this leads to the accumulation of stalled ribosomes. The 3′-OH group of the terminal adenosine of the uncharged tRNA molecules seems to be crucial for the recruitment of the idle RelA enzyme, which is activated through binding to the ribosome [59]. Thereby, the synthesis of the (p)ppGpp alarmone is triggered. Increased levels of (p)ppGpp inhibit exopolyphosphatase enzyme (PPX) activity leading to elevated levels of polyphosphate, which functions as a general activator of the Lon protease. Hence, it seems that perpetuation of the dormant state is ensured through a positive feedback control loop, since the activation of Lon protease causes neutralisation of the hipB antitoxin, thereby triggering the survival response and eventually resulting in the production of higher amounts of the (p)ppGpp alarmone [63].

It is worth noticing that a ΔhipBA deletion strain exhibited a lowered frequency of persister cells in the stationary phase after antibiotic treatment [75], and also a decrease in biofilm formation, even in the absence of antibiotics.

### 2.4. Cell-to-Cell Communication. Two-Component Systems, and Quorum Sensing

In order to effectively monitor critical changes in the external environment and to regulate their internal physiology to adapt to such modifications, bacteria developed intricate signal transduction pathways. Bacteria are endowed with stimuli-responsive abilities due to a specialised signal transduction mechanism relying on the two-component systems (TCS). Moreover, since bacteria in the biofilm mode of life seem to respond as a community rather than just a simple collection of individual cells, cell-to-cell signaling appears as being essential for coordinated behaviour. As a further matter, bacterial cells are also engaged in active cross-talk with the host organism. Experimental evidence has been provided to support the involvement of some TCSs in the regulation of certain gene clusters, thereby promoting biofilm development [76,77]. In general, a TCS consists of a sensor histidine kinase (HK) protein and a corresponding response regulator (RR) protein or domain [76]. Several different TCSs may be found in a bacterial cell, since each particular TCS is able to sense and respond to a specific environmental signal such as pH, nutrient levels, osmotic pressure, redox state, quorum-sensing proteins, and antibiotics [76,78,79].

Quorum sensing (QS) is a bacterial cell-to-cell communication mechanism relying on the production, release, and sensing by bacteria of signaling molecules called autoinducers [80,81]. The amount of the autoinducers (AIs) produced is dependent on cell density. Once the concentration of an autoinducer overcomes a threshold concentration, the transcription of a target gene is activated. Thereby, gene expression is regulated in a cell density-dependent manner, allowing bacteria in a biofilm to gain a competitive advantage in surviving and spreading in natural environments. There are differences between the QS systems used by Gram-positive and Gram-negative bacteria regarding the regulatory components and molecular mechanisms. However, all QS systems share a series of common features [82]. First, bacterial response to the signaling molecules is triggered only when the local concentration of AIs overcomes a certain threshold necessary for detection that can be reached only at high cell densities. Second, all bacteria possess specific receptors located in the cytoplasm or in the plasma membrane specialised in the detection of the AI molecules. Third, AIs detection not only triggers the expression of specific target genes required for cooperative behavior, but also upregulates their own production through a positive feedback autoinduction loop.

In Gram-negative bacteria, the signaling molecules are acyl-homoserine lactones (AHLs) produced by LuxI-type AHL synthases. AHL autoinducers are small free diffusible molecules that pass through the cell membrane and leave the cell. At high cell densities, the local concentration of the AI molecules reaches the detection threshold, and the signal is recognised by the cognate LuxR-type receptors, which are cytoplasmic transcription factors. In the absence of the corresponding AIs, most LuxR proteins fail to fold properly and are degraded. However, by binding to the cognate AHL signaling autoinducers, LuxR receptors become stable, dimerise, and bind to DNA, resulting in QS-mediated gene regulation [77].

On the other hand, Gram-positive bacteria use oligopeptides as AIs (AIPs). AIPs produced by ribosomes undergo post-translational modifications before being exported out of the cell by a membrane-associated ATP-binding cassette (ABC) transporter [83]. Further detection and response to AIPs rely on a TCS signal transduction system. The detection of extracellular AIPs is achieved by a membrane-bound sensor kinase protein, which autophosphorylates at specific conserved histidine residues on HK. Next, the phosphoryl group is shuttled either intermolecularly to a conserved aspartate side chain in a cognate cytoplasmic response regulator protein or intramolecularly on a receiver domain. The phosphorylated RR protein is now activated, binds to the target promoter, and modulates gene expression [83].

QS certainly plays an essential role in biofilm development. Nevertheless, it also seems that QS contributes to the enhanced biofilm recalcitrance towards antimicrobials, since biofilms formed by a *P*. *aeruginosa* ΔlasR ΔrhlR strain, which is a QS-deficient mutant were much more sensible towards tobramycin compared with the wild-type strain [83]. The precise mechanisms underlying the QS-mediated amplification of recalcitrance are not elucidated. Hazan et al. [84] demonstrated an intriguing auto poisoning mechanism leading to biofilm formation and antibiotic tolerance in *P*. *aeruginosa*. They showed that the QS-regulated pro-oxidant molecule 2-n-heptyl-4-hydroxyquinolone-N-oxide (HQNO) inhibits the cytochrome *bc1* complex, thereby disturbing the normal electron flow through the respiratory chain. Electrons that would normally be transferred to cytochrome *c*, the next complex in the ECT (electron transport chain), are passed directly to the final electron acceptor, the oxygen molecule, resulting in the subsequent massive production of ROS, which causes disruption of the cell membrane, cell autolysis, and DNA leakage from cells. As previously shown in Section 2.1.2, e-DNA plays an important role in controlling the viscoelastic properties of the biofilm and promotes antimicrobial tolerance.

### 2.5. Mutational Resistance and Gene Transfer

So far, we discussed the triggering factors and the molecular mechanisms leading to changes in gene expression responsible for the phenotypic shift that occurs in biofilm bacteria and renders them more recalcitrant to antimicrobials than their genetically identic free-floating counterparts. However, we should not forget that independently of biofilm formation, planktonic antibiotic susceptible bacteria may select antibiotic-resistant mutants when exposed to subinhibitory antibiotic concentrations. This type of mutational resistance addresses the antibiotic mode of action through one of the following mechanisms:

*(i)*  modifications of the antibiotic target for instance by decreasing its drug affinity [85];
*(ii)*  modification of the drug itself by the expression of drug-inactivating enzymes, such as *β*-lactamases [86];
*(iii)*  decrease in the drug uptake through reduced porin expression [86,87,88,89];
*(iv)*  over-expression of bacterial drug efflux pumps thereby extruding the antibiotic out of the bacterial cell [12,90,91,92];
*(v)*  development of alternative metabolic routes that bypass those pathways targeted by the antibiotic [93].

Bacteria that have previously undergone mutations can also associate to form biofilms, and biofilm association enhances the mutability rate [94]. The dense and packed structure of biofilms promotes horizontal gene transfer—that is, the transfer of genetic material between cells except for transfer from parent to offspring. HGT can be accomplished through a transformation in which bacteria uptake naked DNA from their environment through conjugation, which requires cell-to-cell contact presumably via pilus, or through transduction when DNA is carried from the donor to the receiver cell by a viral vector or bacteriophage.

## 3. Nanotechnology Solutions for Biofilm Control

**Figure 1:**
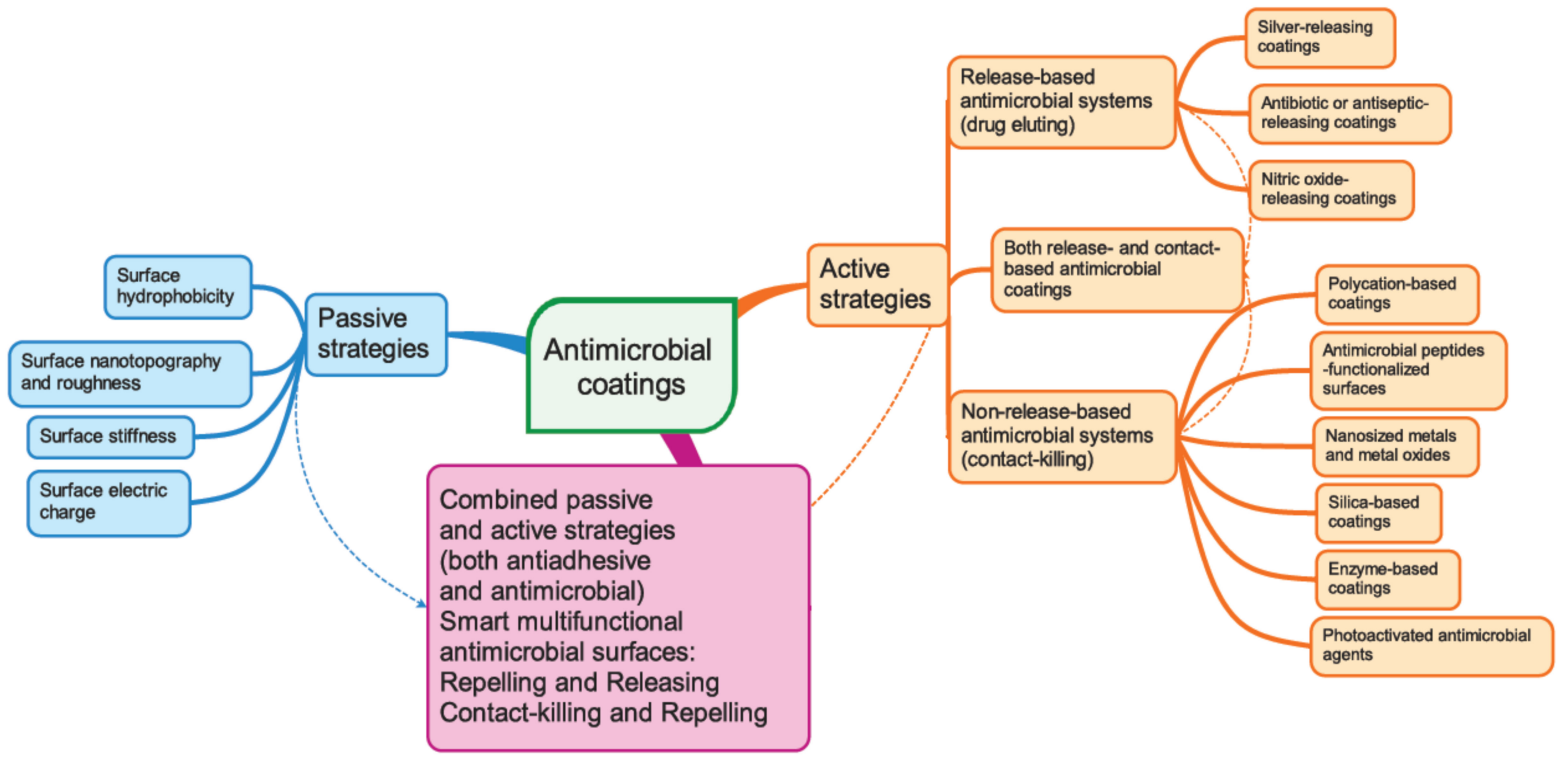
Schematic representations of various currently used strategies to design anti-infective nanostructured surfaces.

Nanotechnology offers promising solutions and innovative strategies for overcoming the problem of biofilm resistance and tolerance [7]. Based on our present knowledge of biofilm properties and formation mechanism, three main strategies to combat biofilm development emerged (see Figure 1): passive strategies, active strategies, and a combination of these two strategies. All these strategies involve various modifications of the surface properties of the implanted biomaterial or its bulk chemistry.

The passive concept aims to prevent adhesion by using antifouling coatings that inhibit the adherence of microorganisms without interfering with the proliferation or the metabolism of the microorganisms. Bacterial attachment can be impeded by controlling the surface hydrophobicity, the surface nanotopography and roughness, the surface stiffness, and the surface electrostatic charge [95].

The active concept uses coatings with intrinsic antimicrobial properties. Two different strategies fall in this category, i.e., non-release-based antimicrobial systems that are thought to act by contact killing and release-based (drug-eluting) coatings [95,96]. The first one relies on the covalent immobilisation of the biocidal compounds onto biomedical device surfaces, rendering them antimicrobial properties. The second strategy is based on physical immobilisation of the biocides into matrix coatings from which after implantation, the biocides are gradually released into the surrounding environment through various mechanisms such as bulk and surface erosion, diffusion, and osmotic pressure. Different encapsulation techniques such as solvent displacement, emulsion-solvent diffusion, emulsion-solvent evaporation, salting out, coacervation, and layer-by-layer assembly can be used to incorporate the active ingredients into the antimicrobial coatings. The antibacterial activity may rely on different mechanisms and compounds: polycations that are able to disrupt the bacterial cell wall allowing the cell contents to spill out; antimicrobial peptides; antimicrobial enzymes that are able to degrade the chemical constituents of the biofilm matrix (DNA, polysaccharides, proteins); antibiotics that interfere with microbes vital metabolic processes such as cell respiration, cell division, or cell wall synthesis; antiseptics; and QS inhibitors that interfere with the bacterial sensing network [96,97]. Antimicrobial coatings targeting inhibition of the transition of the planktonic phenotype of bacteria into a sessile type represent another promising strategy [98]. Multifunctional coatings that combine the above two approaches exhibiting both antiadhesive and antimicrobial properties have also been designed.

The present review given in two parts will highlight the most recent and significant achievements in the field of antimicrobial coatings, emphasising the functional principles, the synthetic strategies, and the antibacterial and antifouling performances of these materials. Smart stimuli-responsive and multifunctional materials are also included.

## 4. Passive Anti-Biofouling Nanocoatings

Passive antibiofilm nanocoatings target the early stage of biofilm formation that is bacterial adhesion onto the surface, which is very important, but they do not kill bacteria. The nanoengineering of coatings falling in this category aims to alter the physicochemical properties and/or topological features of the substrate surface in such a way that the bacteria-substrate interactions become unfavourable. However, the efficacy of this approach depends on the type of adhering bacteria, on the type of the substrate, and on the environmental conditions. According to the actual antibiofouling mechanism, passive strategies can be further subdivided into two categories: fouling resistance strategies and fouling release strategies, respectively [99]. Biofouling can be effectively prevented either through the surface modification of biomedical devices by coating them with various types of polymers such as hydrophilic polymers, zwitterionic polymers, hydrophobic polymers, or via the creation of bioinspired low-adhesion superhydrophobic self-cleaning surfaces by engineering surface nanotopography and roughness [100].

### 4.1. Fouling Resistance Strategies. Hydrophilic and Zwitterionic Polymers

“Fouling resistance” aims to prevent the attachment of biofoulants onto the substrate by suppressing the non-specific non-covalent intermolecular forces, developing when an incoming bacterial cell approaches the substrate surface. The main “fouling resistance” materials are polyethylene glycol (PEG), zwitterionic polymers, glycomimetic polymers [101], and peptidomimetic polymers [102]. There are two main mechanisms underlying the “fouling resistance” strategy: (1) the steric repulsion effect and (2) the formation of a hydration layer [99]. The steric repulsion effect relies on the unfavourable entropy lost originating in the restriction of the free motility of the polymer chains, which are compressed by the foulants during the initial attachment stage [103]. On the other hand, the second mechanism responsible for the antifouling effect of biofouling-resistant coatings involves the formation of a hydration shell through hydrogen bonding in hydrophilic polymers such as PEG or through ion–dipole interactions in the case of zwitterionic polymers. The adsorption of biofoulants requires disruption of the strong associations between the polymer chains and water molecules, which is energetically disfavoured, thereby accounting for the observed antifouling effect [104].

**Figure 2:**
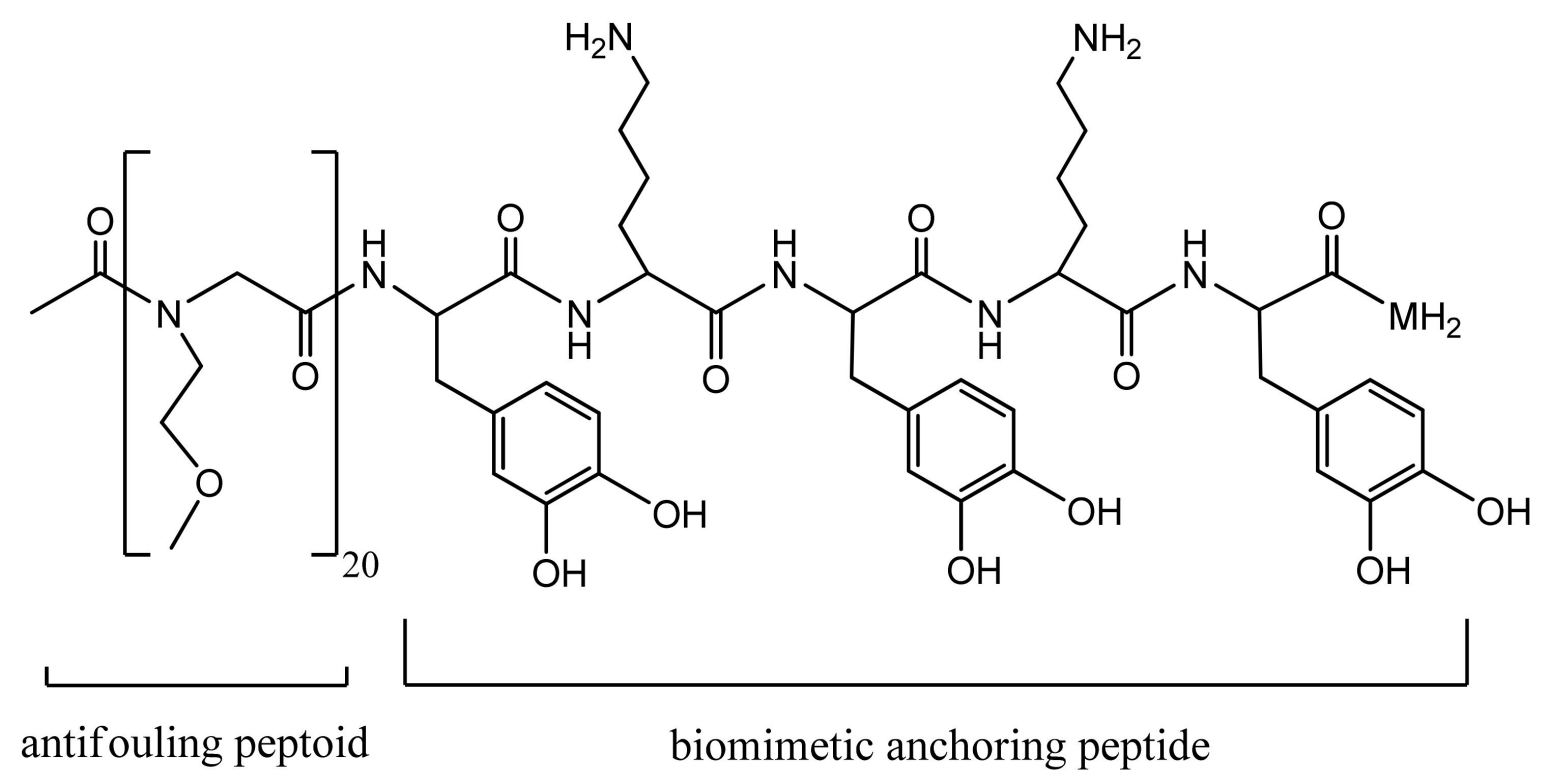
Antifouling peptidomimetic polymer [102]. Reprinted with permission from The New Peptidomimetic Polymers for Antifouling Surfaces. Copyright (2005) American Chemical Society.

For instance, Statz et al. designed a peptide–peptoid polymer conjugate composed of an antifouling peptoid chain and a biomimetic anchoring peptide. The antifouling peptoid portion consists of a glycine polymer *N*-substituted with a methoxyethyl side chain resembling the polar repeating units of the hydrophilic PEG polymer, as shown in Figure 2 [102]. The C-terminal surface anchoring peptide was inspired by the mussel adhesive proteins (MAPs), enabling marine mussels to attach to underwater surfaces strongly. Thus, this surface anchor is composed of the following amino acid sequence: L-3,4-dihydroxyphenylalanine (DOPA)–lysine (Lys)–DOPA–Lys–DOPA. The designed bioinspired peptidomimetic polymer exhibited strong antifouling properties resisting mammalian cell attachment for more than five months [102].

**Scheme 1:**
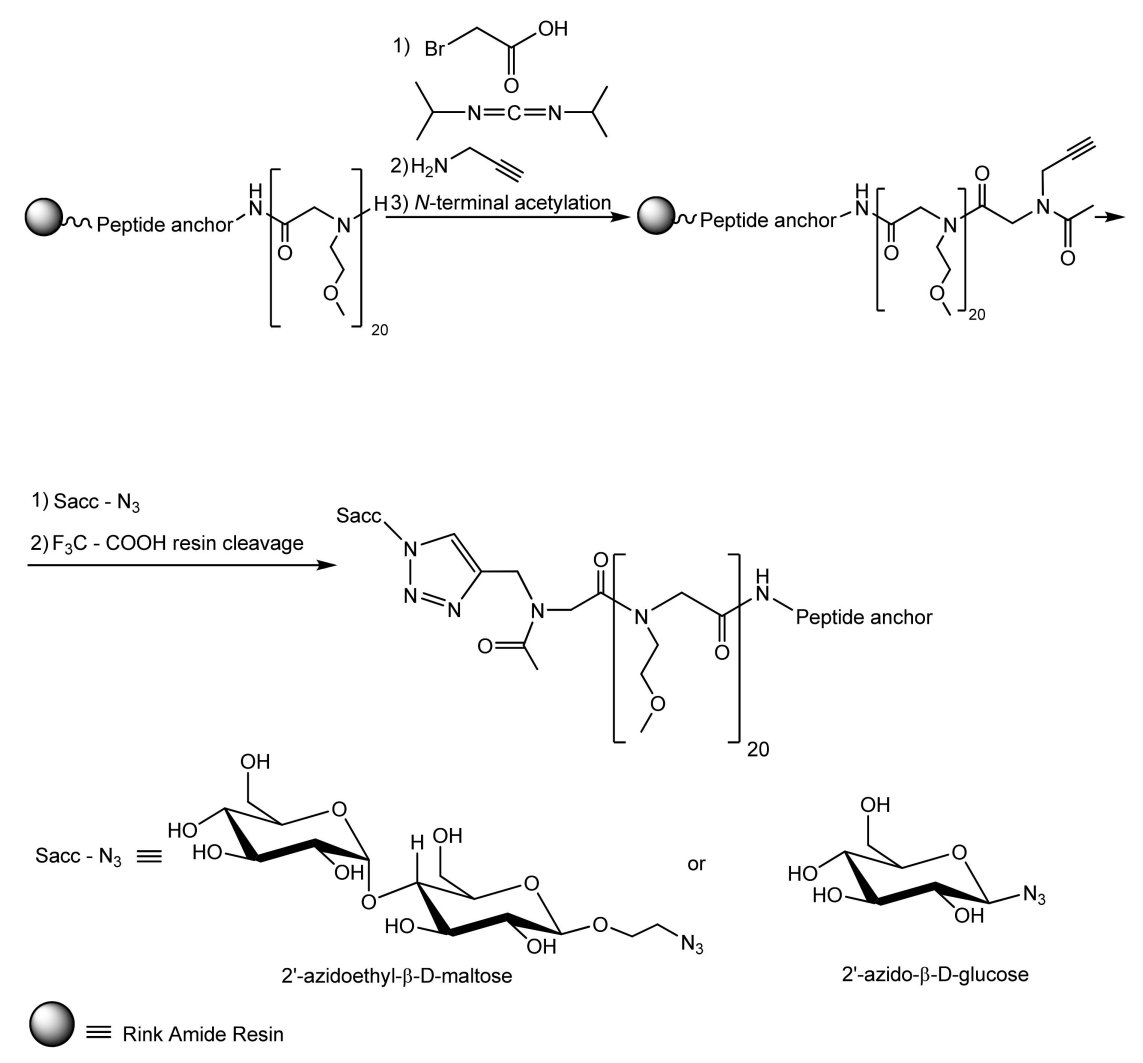
Syntehsis of glycocalyx-mimetic antibiofouling polymer [105]. Reprinted with permission from Antifouling Glycocalyx-Mimetic Peptoids. Copyright (2013) American Chemical Society.

Ham et al. [105] ingeniously used the same peptide anchor to graft glycopeptoids on TiO_2_ support chosen as a representative biomaterial that commonly covers the surface of the titanium alloys largely used in many indwelling medical devices. The authors built up the peptide anchor and the antifouling PEG-mimetic peptoid segments through the well-known solid phase peptide synthesis method. Next, the peptoid segment was modified so as to enable coupling to a hydrophilic saccharide moiety via the extremely versatile click chemistry technique, as shown in Scheme 1. The resulted glycocalix-mimetic polymer drastically increased resistance to non-specific fibrinogen adsorption, fiboblasts adhesion, and bacterial colonisation. The authors concluded that the increased fouling resistance of grafted glycopeptoids compared to bare peptoids is the consequence of a combined effect resulting from the higher number of hydrogen bonds established between water molecules and the interfacial saccharide moieties, which impede interactions with biomolecules and from the steric hindrance of the polymer backbone [105].

The most common hydrophilic polymer widely used in the antifouling surface modification is PEG. The PEG chains are highly hydrated, resulting in a steric hindrance towards bacterial cell attachment derived from the compression of the hydrated layer occurring when bacteria approach the surface, which in turn decreases the conformational entropy of the polymer chains and develops a repulsive elastic force [106,107]. Lichter et al. [108] experimentally demonstrated the direct correlation existing between the substrata stiffness and the adhesion of viable bacteria: the higher the elastic modulus of the substrate (i.e., the stiffer the material), the larger the degree of bacterial adhesion.

**Scheme 2:**
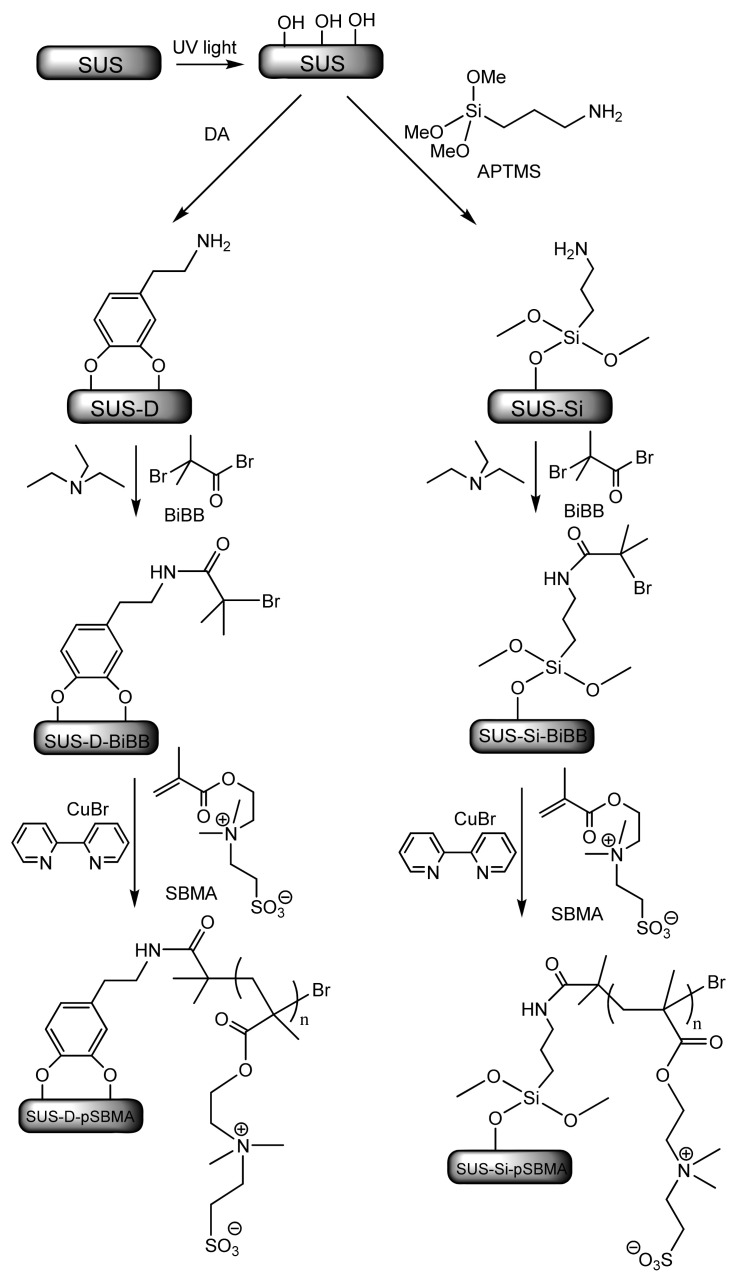
Grafting zwitterionic poly(sulfobetaine methacrylate) (pSBMA) brushes from stainless steel via surface-initiated atom transfer radical polymerization (SIATRP) using polydopamine (PDA) and, respectively, organosilane as self-assembled anchoring agents [109]. Adapted with permission from Zwitterionic-Based Stainless Steel with Well-Defined Polysulfobetaine Brushes for General Bioadhesive Control. Copyright (2014) American Chemical Society.

Due to the very strong electrostatic interactions existing in zwitterionic polymers, they have a very tightly bound hydration layer resulting in “superhydrophilicity” and high anti-bioadhesion properties [100]. Using a grafting from strategy, Sin et al. [109] prepared surface grafted poly(sulfobetaine methacrylate) (pSBMA) brushes by atom transfer radical polymerisation (ATRP). Self-assembled layers of polydopamine (PDA) and organosilane were used as a surface anchor on stainless steel coupons (SUS) for the coupling of pSBMA brushes. The synthetic procedure is depicted in Scheme 2. Cleaned SUS 316L coupons were subjected to UV irradiation for 20 min, followed by cleansing with ethanol and deionised water. For PDA as an anchoring agent, SUS coupons were dipped in buffered ((tris(hydroxymethyl)aminomethane or TRIS) aqueous dopamine solution followed by rinsing with ethanol and deionised water to remove excess unbound dopamine. For organosilane as an anchoring agent, SUS coupons were immersed in a solution of 3-aminopropyl trimethoxysilane (APTMS) in hexane at 40 °C for 2 h, followed by cleansing with ethanol and deionised water. Next, SUS 316L coupons were reacted with 2-bromoisobutyryl bromide (BiBB) under an inert atmosphere, in the presence of the hydrobromic acid scavenger, triethylamine, when the self-assembled anchor layers of the ATRP initiators SUS-D-BiBB, and SUS-Si-BiBB, respectively, were obtained (see Scheme 1). Eventually, ATRP of [2-(Methacryloyloxy)ethyl]-dimethyl(3-sulfopropyl)-ammonium hydroxide (SBMA) was carried out for 24 h, in the presence of Cu(I) bromide as a catalyst, and of 2,2′-bipyridine as a ligand, with deoxygenation (under nitrogen stream). The prepared grafted zwitterionic polymer brushes SUS-D-pSBMA and SUS-Si-pSBMA showed good antibiofouling properties being able to resist fibrinogen, blood platelets, and leukocytes adsorption. Hence, they are promising candidates for thrombogenic coatings. Moreover, only 0.8% *E*. *coli* adhesion and 0.02% *S*. *epidermidis* adhesion were observed on SUS-D-pSBMA [109].

**Scheme 3:**
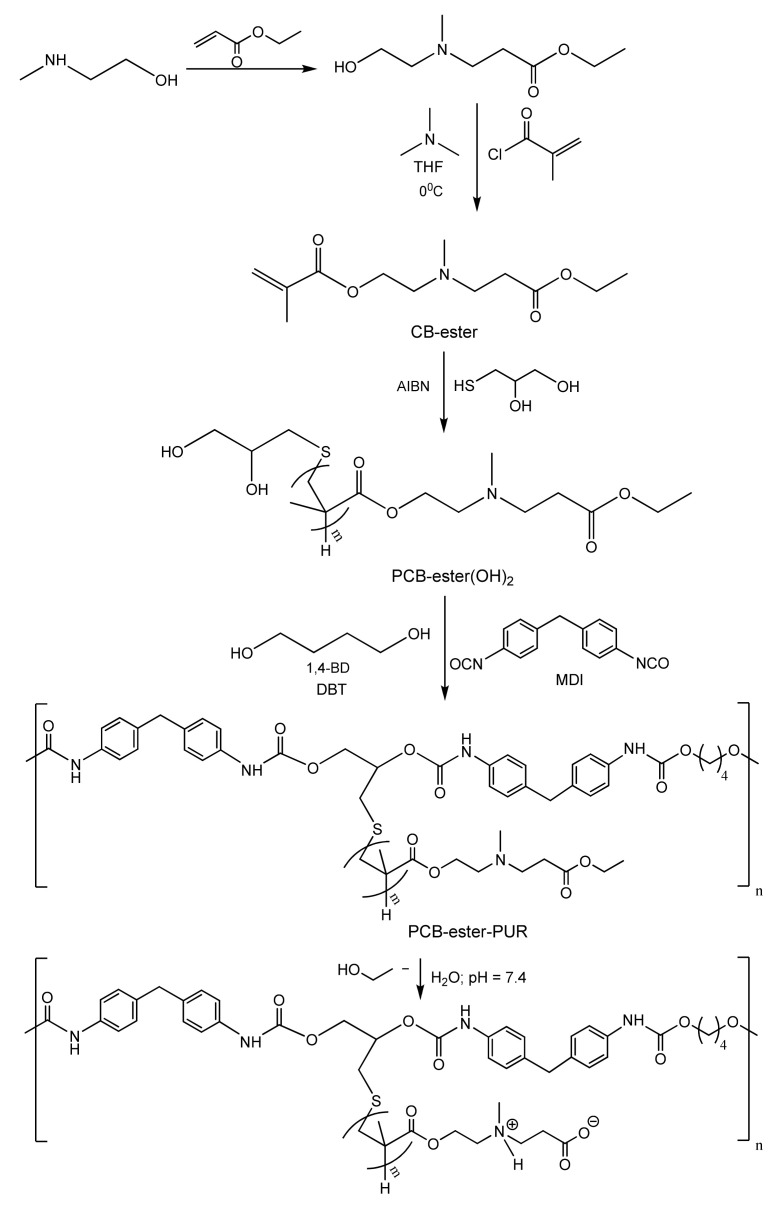
Synthesis and hydrolysis of polyurethane (PUR) brushes with poly(carboxybetaine) ester analog side chains [110]. Adapted with permission from the Development of Robust and Recoverable Ultralow-Fouling Coatings Based on Poly(carboxybetaine) Ester Analogue. Copyright (2015) American Chemical Society.

Antiadhesive polymer brush coatings with higher mechanical strength and ability to self-regenerate have been prepared by Wang et al. [110] based on hydrophobic polyurethane with hydrophobic poly(carboxy betaine) esters as side chains, which can be converted into the corresponding nonfouling zwitterionic poly(carboxybetaines) through hydrolysis. The advantage of this approach resides in combining in one material the mechanical strength and hardness imparted by the hydrophobic polymer matrix with the antifouling and self-healing abilities brought by the zwitterionic poly(carboxybetaine) segments formed at the outer surface of the polyurethane film through the hydrolysis of the poly(carboxybetaine) ester side chains. The synthetic pathway comprises three steps: (1) the synthesis of the carboxybetaine ester analog (CB-ester), (2) the synthesis of the dihydroxy-terminated poly(carboxybetaine) ester analog (PCB-ester(OH)_2_) side chains, and (3) the synthesis of polyurethane with PCB side chains (PCB-ester-PUR). The first synthetic step involves the Michael addition of 2-(methylamino) ethanol to ethyl acrylate followed by the reaction of the resulted intermediate addition product with methacryloyl chloride at 0 °C, in the presence of triethylamine (see Scheme 3). The second step consists in the free radical polymerisation of CB-ester in the presence of azobisisobutyronitrile (AIBN) as an initiator and of 3-mercapto-1,2-propanediol as a chain transfer agent to control the polymerisation degree, m. The stepwise polycondensation reactions of the macrodiol PCB-ester(OH)_2_ with 4,4′-diphenylmethane diisocyanate (MDI) and 1,4-butanediol (1,4-BD) as a chain extender complete the synthesis. The eventual polycondensation reactions were carried out under nitrogen atmosphere. At the same time, chain extension of NCO-terminated prepolymers was performed in the presence of dibutyltin dilaurate (DBT) as a catalyst. Hydrolysis of the hydrophobic PCB-ester side chains on the surface of the polymer brush matrix at the physiological pH of the blood plasma converts them into zwitterionic poly(carboxybetaines), rendering the surface resistant to protein adsorption and bacterial cell attachment for a long period. By hydrolysis, the surface of the polymer brush coatings is regenerated and its nonfouling properties are restored, thereby releasing all attached microbes as proved by the strong inhibitory effect on bacterial and human umbilical vein endothelial cell (HUVEC) attachment as well as on non-specific protein adsorption [110].

### 4.2. Fouling Release Strategies

“Fouling release” strategies aim to drive away the biofoulants already attached to the surface. It was inspired by the self-cleaning properties of the natural epicuticular wax of lotus leaves [111] and envisaged a weakening of the non-specific interactions established between the biofoulants and the depositing surface so that the attached foulants can be easily removed at low hydrodynamic shear forces [112]. This strategy needs the construction of surfaces with low surface energy and a low Young’s modulus from polymeric materials such as silicone or fluorocarbon-based polymers [113].

#### 4.2.1. Hydrophobic Polymers

Using the SIATRP technique (Surface-initiated atom transfer radical polymerization), Xue et al. recently prepared superhydrophobic surfaces by grafting fluorinated polymethacrylates from previously chemically-etched polyethylene terephthalate (PET) fabrics [114]. The authors reported the excellent antifouling abilities and robustness for their superhydrophobic fabrics, which preserved these properties even after 2500 abrasion cycles, 1000 laundry cycles, and prolonged exposure to UV irradiation. Since PET is one of the most largely used plastic materials in medical applications in the form of membranes, filaments, and meshes including surgical meshes, vascular grafts, sewing cuffs for heart valves, and scaffolds for ligament and tendon repair [115], the improvement of the antifouling properties of PET-based medical devices is of course of great interest.

#### 4.2.2. Biomimetic Hierarchical Micro- and Nanostructured Superhydrophobic Surfaces with Low-Adhesion and Self-Cleaning Properties

Wetting can be described as the displacement of a gaseous phase (in particular air) at the surface of a solid by a liquid phase (in particular water).

The wettability of a solid surface by a liquid is described by the contact angle (*θ*), which is the angle at which the liquid–air interface meets the solid–liquid interface. Hydrophilic surfaces are characterised by contact angles to water lower than 90°, while if the water contact angle is higher than 90°, the surface is said to be hydrophobic. In the case of an ideal flat (smooth) surface, the contact angle (*θ*_0_) is given by the Young Equation (1):

(1) $$\cos\theta_{0}=\frac{\gamma_{\mathrm{SA}}-\gamma_{\mathrm{SL}}}{\gamma_{\mathrm{LA}}}$$

where *γ*_SA_, *γ*_SL_, and *γ*_LA_ are the surface tensions at the solid–air, solid–liquid, and liquid–air interfaces.

On a real rough surface having roughness details smaller in size than the liquid droplets, the actual contact angle is the angle between the tangent to the liquid–air interface and the actual local surface of the solid. However, the actual contact angle is usually not a measurable quantity, and what is currently amenable to measurement is the apparent contact angle defined as the angle between the tangent to liquid–air interface and the apparently solid surface, as macroscopically observed. The apparent or measured contact angle θ is often modeled using either the Wenzel’s Equation (2) or the Cassie–Baxter Equation (3) depending on whether the liquid penetrates or not into the grooves of the rough solid surface [116,117,118,119,120].

**Figure 3:**
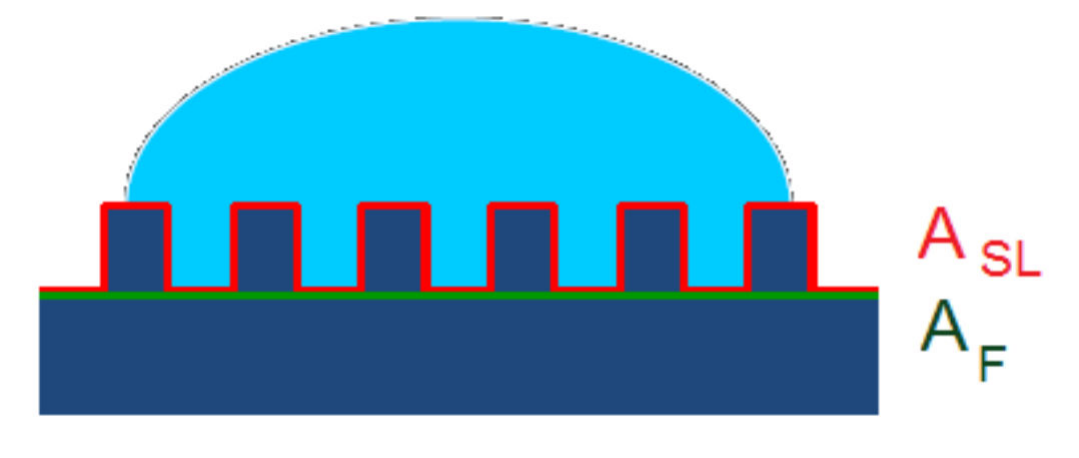
The Wenzel model presumes homogenous wetting. The surface below the droplet is completely wetted by the liquid.

Wenzel’s model assumes that the liquid droplet fills up the grooves completely on a rough surface, forming a homogenous interface with the solid (see schematic representation in Figure 3). According to Wenzel, the apparent contact angle (*θ*) can be related to the Young contact angle on an ideal smooth surface (*θ*_0_) by Equation (2). The proportionality constant *R*_f_ in Equation (2) is called the roughness factor, which accounts for the extension of the surface area due to the roughness being defined as the ratio of the actual surface area of the liquid–solid interface (*A*_SL_) to the geometrically projected area, or in another word, the flat solid–liquid contact area (*A*_F_).

(2) $$\cos\theta =R_{f}\cos\theta_{0}\;$$

$$R_{f}=\frac{A_{SL}}{A_{F}}$$

The Wenzel Equation (2) predicts that the wettability caused by the chemistry of the surface is reinforced by roughness. Since the roughness factor is always supraunitary, R_r_ > 1, if the solid surface is mostly hydrophilic, i.e., if *θ*_0_ < 90°, and cos*θ*_0_ > 0, then cos*θ* > cos*θ*_0_, meaning that *θ* < *θ*_0_. On the other hand, if the solid surface is mostly hydrophobic (*θ*_0_ > 90°, cos*θ*_0_ < 0), then cos*θ* < cos*θ*_0_, which means that *θ* > *θ*_0_. In other words, a hydrophilic surface becomes even more hydrophilic, and a hydrophobic surface becomes even more hydrophobic if the roughness increases. However, in practice, the Wenzel model is mostly applicable to hydrophilic surfaces, since, on rough hydrophobic surfaces, the liquid cannot wet the whole surface [121]. In fact, Equation (2) is valid only for moderate values of the roughness factor, *R*_f_.

**Figure 4:**
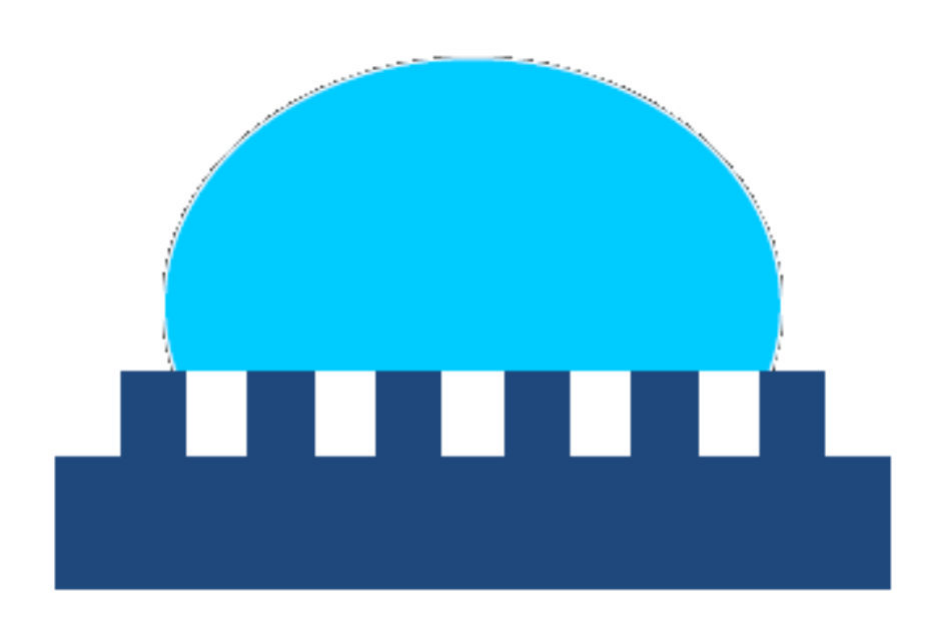
The Cassie model presumes that in the grooves, the air is trapped below the liquid droplet resulting in a composite solid to liquid-to-vapor interface.

For non-wetting surfaces, as the roughness increases, it becomes harder and harder for the water to penetrate into the grooves, resulting in the partial wetting and formation of trapped air pockets between the liquid and the rough solid surface (see Figure 4). That leads to a composite interface consisting of solid–liquid, solid–air, and liquid–air interfaces where the solid–air interfaces and the liquid–solid contact zones are located into the troughs of the rough surface and, at the top of the asperities, respectively. In this situation, increasing the surface roughness will no longer result in an increase of the solid–liquid contact area. For the case of partial wetting and considering a flat composite interface under the droplet, the apparent contact angle θ is given by the Cassie–Baxter Equation (3).

(3) $$\cos\theta =R_{f}f_{\mathrm{SL}}\cos\theta_{0}-f_{\mathrm{LA}}=R_{f}f_{\mathrm{SL}}\cos\theta_{0}-1+f_{\mathrm{SL}}$$

where *f*_SL_ and *f*_LA_ are the flat geometric areas of the solid–liquid and liquid–air interfaces, respectively.

The Cassie–Baxter Equation (3) predicts that for higher roughness (lower pitch between the asperities), the solid–liquid contact area dramatically decreases, *f*_SL_ approaches zero, and θ approaches 180°. Hence, the surface becomes superhydrophobic; the adhesion of the liquid to the solid is greatly reduced, which aids a droplet on the surface to roll off easily even at a low inclination. Moreover, the adhesion of the dirt particles to such a surface is minimal because of the very small contact area between the particles and the rough surface. Hence, if the rolling liquid droplet encounters a particle lying on the surface, it readily picks up the particle because the contact area between the droplet and the particle is much larger than that one between the particle and the rough solid surface. Consequently, the dirt particle will adhere to the liquid droplet, being carried away from the surface by the rolling drop. This is the underlying mechanism behind the well-known self-cleaning ability of the *Nelumbo nucifera* (Lotus) leaves.

In conclusion, the formation of a stable composite interface with air pockets between the liquid and the solid appears to be of critical importance in order to attain superhydrophobicity and self-cleaning properties.

**Figure 5:**
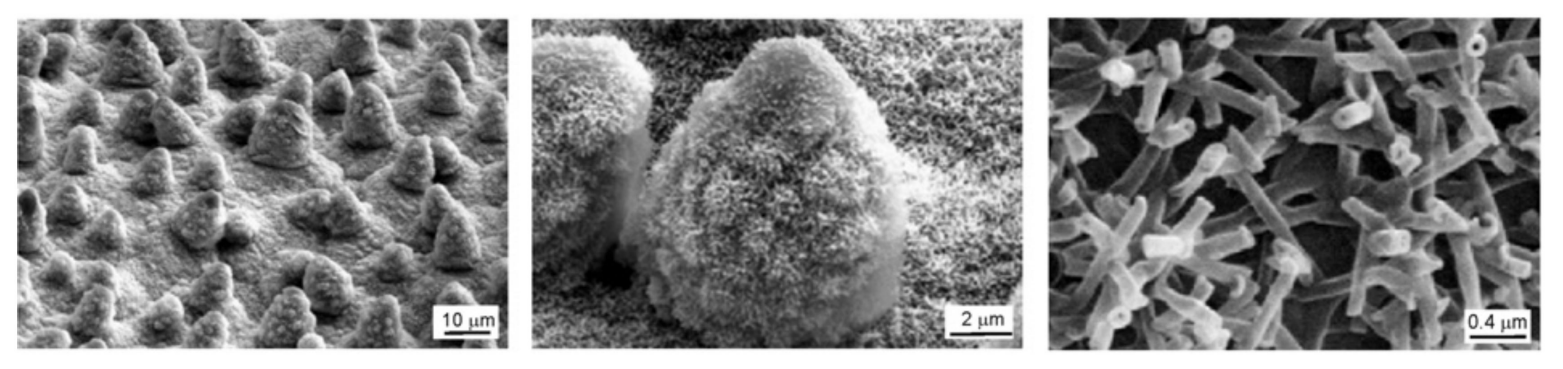
Scanning Electron Microscopy (SEM) micrographs of Lotus leaf revealing a hierarchical dual-scale roughness. Reproduced from reference [116] with the permission of the Royal Society of Chemistry. Copyright (2009) Royal Society of Chemistry.

However, the composite interface is fragile, and a series of destabilising factors such as the formation of capillary waves at the liquid–air interface, nanodroplet condensation, as well as liquid pressure can irreversibly transform it into a homogenous interface [118,119,122,123]. SEM (Scanning Electron Microscopy) micrographs revealed the peculiar hierarchical surface morphology of the Lotus leaves. The upper epidermis of a Lotus leaf has a distinctive hierarchical structure consisting of micro-bumps of epidermal cells (papillae) coated with a dense covering of agglomerated nano-scale epicuticular wax tubules [111] (see Figure 5 for SEM micrographs of Lotus leaf at three magnifications). It is this combination between the hydrophobic coating and the peculiar surface topography with dual hierarchical roughness on micro- and nano-length scales that are responsible for the Lotus effect. These specific topographical features enhance the stability of the composite interface, as demonstrated by Nosonovsky and Bhushan. These authors showed that the higher micro-scale papillae resist the capillary waves, while nanoscale epicuticular tubules prevent nanodroplets from filling the grooves between the papillae [118].

There are several methods to fabricate biomimetic superhydrophobic surfaces with hierarchical roughness. They can be categorised as “top–down” or “bottom–up” approaches. In the top–down approaches, the nano-scale topographical details of the surface are obtained by the gradual removal of material from a larger bulk substrate [124]. From the top–down fabrication techniques, we mention: (1) templating, which is a replication technique allowing the creation of a surface topography that is a complementary (negative) replica of an appropriate specially prepared template [125,126], (2) lithography, including photolithography, electron beam lithography, and ion beam lithography which all share the principle of transferring an image from a mask to a receiving substrate [127], (3) chemical and plasma etching [128,129], (4) anodic oxidation [130], and (5) laser ablation [131]. The bottom–up approach is exactly the opposite of the top–down approach, since it starts with atoms and molecules and builds up to larger nanostructures. In a bottom–up structuring process, the micro/nanotopographic features of the surface are created by the controlled sequential deposition of material onto a substrate. The deposition techniques can be based on (1) purely physical processes such as evaporative methods, physical vapor deposition, and electrospinning [132,133]; (2) physicochemical processes such as molecular self-assembly [134,135], and (3) chemical processes. The chemical deposition processes involve the formation of a thin solid film on a substrate by a chemical reaction of some precursors that may initially be either in a solution such as in the sol–gel methods [135,136], the hydro- or solvothermal syntheses [137], and electrochemical deposition [138,139,140], or in the vapor phase such as in the chemical vapor deposition (CVD) method [141].

Ho et al. [142] reported two cost-effective approaches to the fabrication of superhydrophobic polymer films with biomimetic Lotus-like hierarchical topography and self-cleaning properties using the templating technique. In one strategy, the first hierarchical level of the template consisting of a two-dimensional array of silicone microdomes was created through lithographic surface patterning. Next, the substrate was covered with a thin film of Ti, followed by the electron beam physical vapour deposition of an aluminum film 1 µm thick. The second hierarchical level was achieved through surface nanotexturing the aluminum film by anodisation and etching. It is known that when anodised in an acidic medium, aluminium oxidises to form a nanoporous alumina layer [143]. The nanopores were further broadened to 200 nm by chemical etching with a solution of phosphoric acid. A negative replica of the so-prepared silicon-anodised aluminium oxide (AAO) mold was fabricated by nanoimprint lithography [144]. In order to reduce the surface energy and to facilitate demolding, the silicon–AAO template was first subjected to CVD of 1H,1H,2H,2H-perfluorodecyl trichlorosilane. Then, a 500 µm thick film of polypropylene (PP) was placed on the silicone–AAO template and heated to 180 °C with a concomitant application of 60 Bar pressure for 5 min so that the polymer melt could infiltrate into the mold. Next, the polymer–mold assembly was cooled down to a temperature of 90 °C, the pressure was released, and demolding was carried out by simply pilling off the template. In the second approach, the ordered array of microdome structures forming the first hierarchical level of the mold was prepared in an aluminium plate approximately 1 mm thick with the aid of a computer numerical control machine tool. An ion track-etched nanoporous polycarbonate membrane was used as a nanotemplate. The polycarbonate membrane was placed over the aluminium mold. Next, the polypropylene film to be imprinted was placed on the top of the assembly, and heat and pressure were applied. As a consequence, the nanoporous polycarbonate membrane adopted the dome-shaped microtopography of the aluminium mold, while the nanopores were filled up with the molten polypropylene. By this imprinting process, the hierarchical topographical features of the microdome patterned aluminum mold-nanoporous polycarbonate membrane template assembly were copied on the polypropylene film as an inverse replica. After demolding and etching of the polycarbonate membrane, hierarchically structured polypropylene films resembling the Lotus leaves surface topography comprising microdomes having diameters in the range from about 5 to about 400 µm, heights in the range from about 2.5 to about 500 µm, and pillars covering the domes with diameters in the range from about 20 nm to about 5 µm and aspect ratios (height: diameter ratios) in the range from 2 to about 50 were prepared [142,145]. Medical devices coated with such films displayed antifouling properties [145].

Ye et al. [146] developed a new bottom–up approach to the fabrication of superhydrophobic surfaces by casting a solution of an elastomer, styrene-*b*-(ethylene-*co*-butylene)-*b*-styrene (SEBS), on different substrates (glass, polymer, metal) followed by evaporation of the solvent. Unlike most polymers used to fabricate superhydrophobic surfaces by casting methods, which are crystalline or plastic at room temperature, elastomers are amorphous and soft, comparatively presenting a much looser packing and consequently increased mobility of the polymer chains. Hence, the micro- and nanostructures formed during solvent evaporation are not stable and collapse. The novelty of the fabrication method proposed by the authors consists of the utilisation of a mixture of a polymer solvent and a polymer nonsolvent in an appropriate ratio to prepare the casting solution. More specifically, they used decanol and *p*-xylene as a nonsolvent, and respectively, as a solvent for the said SEBS elastomer. Since the nonsolvent has a higher boiling point (233 °C) than that of the solvent (137 °C), it tends to remain in the gel-like body of the elastomer during evaporation of the solvent. Therefore, it can act as a soft liquid template inducing aggregation and stabilising the SEBS micro- and nanostructures newly formed during the evaporation of the solvent. After eventual drying under vacuum, a solid superhydrophobic film having a porous, rough surface morphology was left on the substrate. The distribution and the size of the pores could be tuned by varying the solvent/nonsolvent ratio. The SEBS coatings cast from a 50:50 (*v*/*v*) mixture of *p*-xylene and decanol exhibited a contact angle of 156°. For comparison, the pristine commercial SEBS elastomer with 29 wt.% styrene has a contact angle of only 96°. Moreover, while the original elastomer had no significant antibiofouling properties, the superhydrophobic SEBS surfaces manufactured by this method completely inhibited the adhesion of both *E*. *coli* cells and platelets, as proved by SEM imaging [146]. Furthermore, the superhydrophobic SEBS coatings exhibited improved anticoagulant properties. The blood clotting time indexes (BCIs) on these surfaces had increased up to four times as compared to the corresponding BCIs on the pristine SEBS elastomer. They also showed good biocompatibility, the hemolysis ratio on the SEBS superhydrophobic coatings being up to eight-fold lower than that on the surface of the commercial SEBS elastomer [146].

However, the structured superhydrophobic surfaces in the Cassie–Baxter regime are prone to loss of their antibiofouling properties, especially under submerged conditions when an irreversible transition to the Wenzel state can easily occur. Traditional antibiofouling surfaces are solid materials, meaning that their surface atoms or molecules are fundamentally static in nature, which is a feature that depending on the time scale of the adhesion process, can favour stable attachment and biofouling. Therefore, the creation of a material exhibiting the intrinsic mobility of its surface molecules and structures represents a valuable alternative that might significantly hamper biological adhesion. Keeping that in mind, Epstein et al. [147] came up with a completely different strategy for fabricating antibiofouling surfaces: slippery liquid-infused porous surfaces (SLIPS). The fabrication of SLIPS involves four sequential steps as follows: (1) surface nanopatterning of a flat substrate by any of the top–down or bottom–up approaches such as etching and lithographic techniques or sol–gel processes; (2) chemical functionalisation of the rough nanostructured surface of the substrate in order to increase its affinity for the lubricant fluid; (3) complete wetting of the substrate with the lubricant through capillary wicking; (4) removal of excess unattached lubricating fluid. To increase the stability of SLIPS under submerged conditions, three important requirements should be fulfilled: (1) the affinity of the substrate surface for the lubricant should be higher than that for the surrounding fluid; (2) the lubricant and the surrounding fluid should be immiscible; and (3) the surface of the solid substrate should be roughened so as to increase the surface area for the adhesion of the lubricating fluid. The authors used nanoporous Teflon membranes as substrates and the perfluorinated compounds perfluoropolyethers (Dupont™ Krytox^®^ 100 and 103), perfluorotripentylamine (3M™ Fluorinert™ FC-70), and perfluorodecalin, as lubricating fluids. The so-prepared SLIPS proved excellent ability to prevent the adhesion of pathogenic bacteria and biofilm formation. Using fluorescence imaging and crystal violet assays, the authors showed that the SLIPS platform prevents 99.6% of *P*. *aeruginosa* biofilm attachment over at least a 7-day period, under dynamic low-flow conditions, while other nanostructured superhydrophobic surfaces developed biofilms within hours. Similar high levels of biofilm reduction were also obtained for *S. aureus* (97.2%) and *E*. *coli* (96%) [147].

### 4.3. Combined Passive Strategies

Neither of the two “fouling resistance” and “fouling release” strategies can render antibiofilm coatings with excellent antifouling performances by itself.

**Figure 6:**
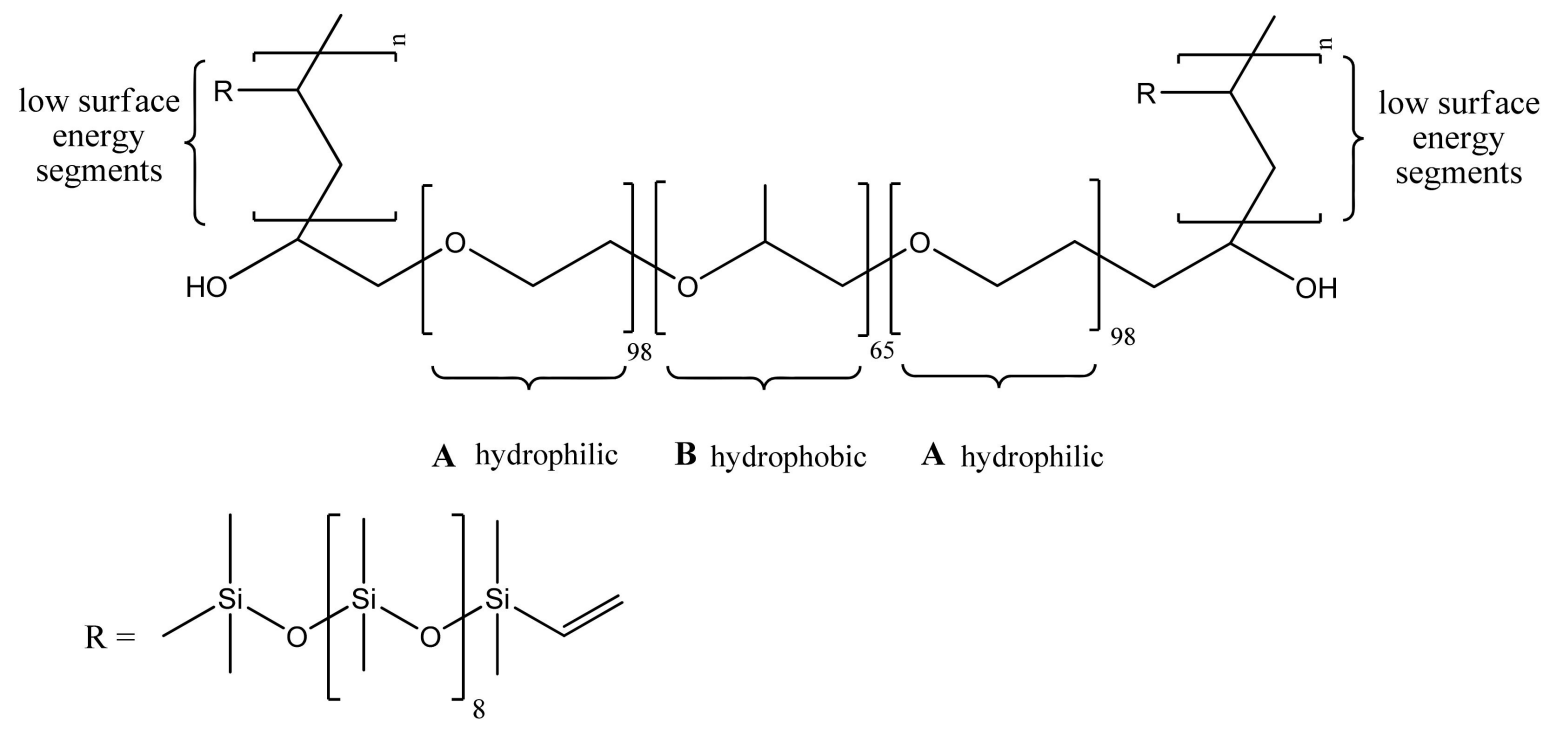
Amphiphilic block copolymer F127-*b*-PDMS_n_ [113]. Reprinted with permission from Comparison of the Fouling Release Properties of Hydrophobic Fluorinated and Hydrophilic PEGylated Block Copolymer Surfaces:  Attachment Strength of the Diatom Navicula and the Green Alga Ulva. Copyright (2006) American Chemical Society. PDMS: polydimethylsiloxane.

Therefore, in order to achieve a synergic antifouling effect, scientists have combined both “fouling resistant” and “fouling release” passive mechanisms in the same unique polymeric nanostructured material. In an illustrative example, Zhao et al. [148] developed an amphiphilic pentablock copolymer by modifying an amphiphilic pluronic F127 triblock copolymer of ABA type composed of two-terminal hydrophilic PEG segments (A blocks) covalently interlinked by a hydrophobic poly(propylene oxide) (PPO) B block. The terminal “fouling resistant” hydrophilic poly(ethylene oxide) blocks were further extended through two covalently attached short low surface energy “fouling release” polydimethylsiloxane (PDMS) segments to obtain an amphiphilic PDMS–PEG–PPO–PEG–PDMS block copolymer, as shown in Figure 6. The amphiphilic F127–PDMS block copolymer was further used to prepare nanocomposite membranes by blending with hydrophobic polyethersulfone (PES). The antifouling membrane was fabricated by the non-solvent induced phase separation method (NIPS) [112]. Generally, during the NIPS process, an amphiphilic block copolymer undergoes spontaneous self-assembly at the water/membrane interface, providing a surface extensively covered by the hydrophilic segments and being anchored with the hydrophobic block to the hydrophobic matrix PES polymer. So, the hydrophilic PEG blocks freely segregate onto the water/membrane interface, which is a process that would be impossible for the non-polar low surface energy PDMS segments due to unfavourable thermodynamics. However, the peculiar chemical structure of the amphiphilic F127–PDMS block copolymer allows for “forced surface segregation” of the hydrophobic non-polar PDMS segments. The short terminal PDMS segments being covalently linked to the spontaneously surface segregated hydrophilic PEG blocks are being dragged by the later rendering the membrane surface with “fouling release” properties in addition to the “fouling resistance” properties imparted by the hydrophilic PEG segments. The described synergic strategy is based on the pioneering work of Wooley. Wooley and co-workers were the first to report the fabrication of antifouling coatings with tunable surface morphologies and topographies based on amphiphilic cross-linked polymeric networks formed in situ from phase-segregating mixtures of incompatible polymeric precursors, namely hydrophobic hyperbranched fluoropolymers (HBFPs) of various polydispersity indexes ranging between 1.8 and 2.4 and hydrophilic bis(3-aminopropyl)-terminated poly(ethylene glycol) [149,150]. The cross-linked network coatings were prepared on glass substrates, functionalised by 3-aminopropyltriethoxysilane. The surface compositions, morphologies, and topographies of the amphiphilic antifouling coatings depend on the degree of cross-linking, which in turn could be controlled by varying the weight ratio between HBFPs and terminal amine-functionalised PEG used for cross-linking with HBFPs through the nucleophilic aromatic substitution of fluorine atoms.

## 5. Conclusions

Along with the development of modern surgical techniques as well as of invasive diagnostic and monitoring tools, nosocomial infections became one of the leading causes of morbidity and mortality [151]. Therefore, preventing surface colonisation of implantable prostheses and indwelling medical devices by dangerous pathogens and subsequent biofilm formation on such surfaces is critically important first of all for the patient’s condition but also for cutting down the financial and social costs of treating healthcare-associated infections. Furthermore, biofilm formation must be disrupted, since in the biofilm lifestyle, bacteria develop microbial community interactions [152]. This cooperative social behavior is regulated and coordinated through QS mechanisms and contributes to biofilm increased recalcitrance towards antimicrobial agents as compared to free-floating planktonic counterparts, which is mainly due to the enhanced rate of mutations and to altered gene expression in biofilms [153]. Therefore, the study and understanding of the complex processes occurring during different stages of biofilm development and the molecular mechanisms underlying these processes are of crucial importance for the discovery and implementation of new and innovative antibiofilm strategies targeting these processes.

**Figure 7:**
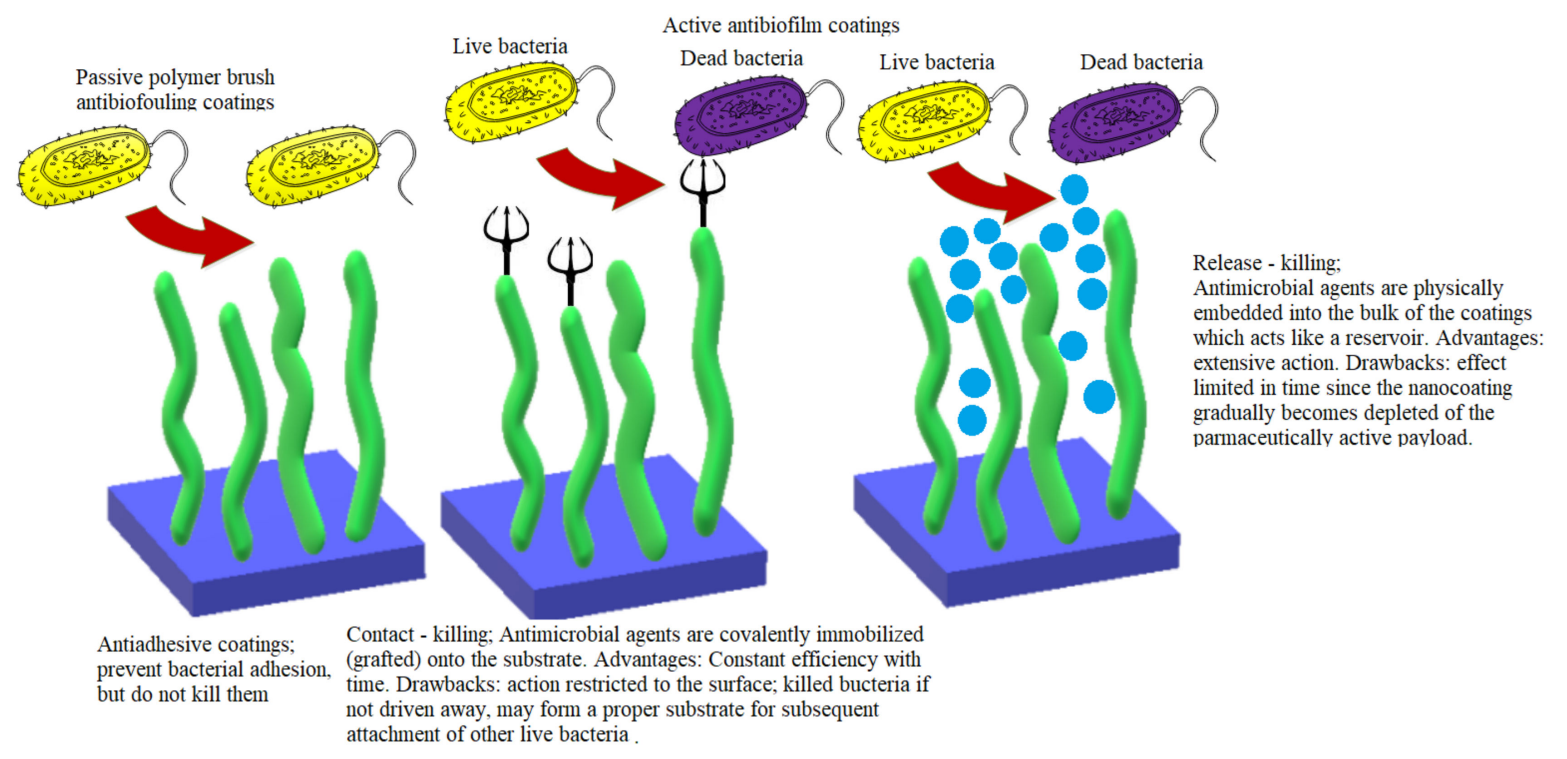
Antibiofilm strategies: advantages and drawbacks.

Over time, scientists developed various nanostructured antibiofilm coatings that can be roughly classified as passive and active. Both strategies have advantages as well as drawbacks, as illustratively depicted in Figure 7 [154].

Although remarkable progress has been made in understanding the bioufouling mechanisms and clear and comprehensible qualitative structure-function/performance relationships such as hydrophilicity— “fouling resistance” and surface energy— “fouling release” have been established, many important aspects still remain to be unveiled. For instance, a precise quantitative relationship between functional moieties and antifouling properties is still lacking. Surface nanoengineering is a powerful tool in manipulating surface chemical composition and functionality, as well as morphology and topography at the hierarchical micro- and nanoscale, in order to achieve higher performances in impeding bacterial adhesion to biotic and abiotic surfaces. Nanotechnology also provides innovative solutions for the practical implementation of multiple antibiofouling mechanisms in a simultaneous synergistic manner.

The active antibiofilm strategies targeting the molecular mechanisms and processes that govern the maturation and dispersal stages of the biofilm life cycle will be addressed in the second part of this review.
